# The Preharvest Application of Stress Response Elicitors Improves the Content of Bioactive Compounds without Modifying the Sensory Attributes of Butterhead Lettuce (*Lactuca sativa* var. *capitata*)

**DOI:** 10.3390/foods13162574

**Published:** 2024-08-17

**Authors:** Laura A. de la Rosa, Jesus Omar Moreno-Escamilla, Nina del Rocío Martínez-Ruiz, Emilio Alvarez-Parrilla, Gustavo A. González-Aguilar, Joaquín Rodrigo-García

**Affiliations:** 1 Departamento de Ciencias Químico-Biológicas, Instituto de Ciencias Biomédicas, Universidad Autónoma de Ciudad Juárez, Av. Benjamín Franklin No. 4650, Zona PRONAF, Ciudad Juárez 32315, Chihuahua, Mexico; ldelaros@uacj.mx (L.A.d.l.R.); jesus.moreno@uacj.mx (J.O.M.-E.); nmartine@uacj.mx (N.d.R.M.-R.); ealvarez@uacj.mx (E.A.-P.); 2 Coordinación de Tecnología de Alimentos de Origen Vegetal, Centro de Investigación en Alimentación y Desarrollo, Carretera a la Victoria km 0.6, Hermosillo 83304, Sonora, Mexico; gustavo@ciad.mx; 3 Departamento de Ciencias de la Salud, Instituto de Ciencias Biomédicas, Universidad Autónoma de Ciudad Juárez, Av. Benjamín Franklin No. 4650, Zona PRONAF, Ciudad Juárez 32315, Chihuahua, Mexico

**Keywords:** elicitation, polyphenols, carotenoids, sensory analysis

## Abstract

Using stress elicitors in fruits and vegetables is considered a good strategy to increase the content of bioactive compounds in plant foods. However, bioactive compounds can affect the sensory characteristics of food products, and little is known about their shelf-life stability in fresh produce treated with elicitors. In the present work, carotenoids and polyphenols were quantified by spectrophotometric methods in red and green butterhead lettuce treated with elicitors that had previously been demonstrated to increase bioactive compounds: arachidonic acid (AA), methyl jasmonate (MJ), and Harpin protein (HP). The bioactive compounds were determined immediately and during three weeks after harvest. A descriptive sensory analysis was carried out, which included odor, taste, tactile, and visual attributes of control and elicitor-treated lettuce. Carotenoids showed greater shelf-life stability than polyphenols, and both were more stable in red than in green lettuce during the first two weeks of storage. The best elicitor was MJ, which increased phenolic compounds (red and green lettuce), anthocyanins, and carotenoids (red lettuce) through the storage period. Color intensity, crispness, wettability, and bitter taste were some of the primary sensory attributes in butterhead lettuce and were not affected by any treatment. Other organoleptic properties were also not affected by the elicitors. These results suggest that elicitation could improve the content of bioactive compounds, which is stable through the shelf-life of butterhead lettuce, without any adverse effect on the sensory properties.

## 1. Introduction

The consumption of a diet rich in plant foods, even a plant-based diet, has attracted significant interest worldwide for health and environmental reasons [1]. Lettuce is among the world’s most consumed vegetables, contributing to this dietary trend. In the United States, lettuce production was above 300 million kilograms between 2016 and 2017 [2]. According to their morphological features, there are four types of cultivated lettuce: romaine, crisphead, butterhead, and loose leaf, and each type has different varieties [3]. Butterhead is a type of lettuce with a small head of loosely folded spherical leaves and an oily texture that makes it attractive to consumers [4]. Most butterhead varieties are greenish, but there are also some red varieties. The major antioxidant compounds in butterhead lettuce are phenolic compounds, carotenoids, and vitamins C and E (tocopherols) [3,5]. The major phenolic compounds identified in butterhead lettuce are hydroxycinnamic acids (mostly caffeic acid derivatives), flavonols (mostly quercetin and kaempferol glycosides), and some hydroxybenzoic acid derivatives; in a red variety, a delphinidine glucoside (anthocyanin) was identified [6]. The major carotenoids are lutein and β-carotene [3]. However, the content of these bioactive compounds is, in general, low compared to other vegetables [7] and even to other lettuce types such as romaine and loose leaf, although fewer studies have been carried out with butterhead than with the other two lettuce types [3]. Antioxidant compounds in plant foods are also known as bioactive compounds because they exert several health-promoting effects on the human consumer. Lettuce and lettuce extracts rich in bioactive compounds (mainly phenolic compounds) have shown cardioprotective, neuroprotective, anticancer, and antidiabetic effects, among others [4]. However, most studies have been conducted in in vitro and animal models [3]. Therefore, different pre- and post-harvest strategies have been developed to increase the content of bioactive compounds and the health-promoting value of lettuce [3,5,6]. Among preharvest treatments, physical or chemical elicitors that induce defense response mechanisms of plants have been used. Plants respond to biotic and abiotic stressors by activating several defense enzymes, including enzymes that synthesize secondary metabolites; the most common are phenolic compounds, which accumulate through the phenylpropanoid pathway [8]. Stress response elicitors such as salicylic acid (SA), methyl jasmonate (MJ), arachidonic acid (AA), and Harpin protein (HP) have been shown to increase the concentration of secondary metabolites, including phenolic compounds, alkaloids, carotenoids, and vitamin C, in butterhead lettuce and other plant foods [9,10,11,12]. An increase in this type of compound is desirable to improve the health properties of vegetables; nevertheless, these compounds may modify the organoleptic characteristics of foods, such as flavor, color, and texture [13]. In a previous study, the effect of different concentrations of four elicitors (MJ, HP, AA, and salicylic acid) and the time of pre-harvest administration on the content of bioactive compounds in green and red butterhead lettuce were studied. Phenolic compounds and carotenoids were increased the most in both lettuce varieties, and the optimal treatments were MJ (90 μM) and HP (60 mg/L) applied seven days pre-harvest in green lettuce, and MJ (90 μM) and AA (45 μM) applied 15 days pre-harvest in red lettuce [5,6]. However, the impact of the increased bioactive compounds on organoleptic characteristics was not determined. Moreover, the shelf-life stability of the bioactive compounds induced by treatment with elicitors has not been studied in detail [12]. For this reason and based on the results obtained in our previous investigations [5,6], this study aimed to evaluate the effect of elicitation with MJ and HP in green lettuce and MJ and AA in red lettuce on the sensory characteristics and shelf-life stability of phenolic compounds and carotenoids.

## 2. Materials and Methods

### 2.1. Plant Materials, Growing Conditions, and Elicitor Administration

Butterhead lettuce plants (*Lactuca sativa* L. var. *capitata*) of green (FVM02 seed) and red (FRM02 seed) varieties were grown hydroponically (January–February 2018) in a recirculating system in a greenhouse with an average photoperiod of 10 h/day under the conditions of 25–28 °C, 40–60% relative humidity, and 35% sunlight blocking at InnoBio Hidroponia Inc. (Chihuahua, Mexico) Mineral nutrients consisted of N (16%), P (4%), K (17%), and a stage II micronutrient solution mix (InnoBio Hidroponia Inc.; pH 6.8, EC = 1800 mS). Based on previous investigations [5,6], MJ (90 μM) and HP (60 mg/L) were selected as elicitor treatments for green lettuce, and MJ (90 μM) and arachidonic acid (45 μM) for red lettuce. Treatments were applied seven days before harvest for green lettuce and fifteen days before harvest for red lettuce based on the response observed in our previous investigation [5]. Each treatment group consisted of 15 green or red lettuces treated with foliar aspersion (three sprays, approximately 1.70 mL per plant). Lettuces were harvested 60 days after planting and stored at 4 °C until further evaluation.

### 2.2. Evaluation of the Stability of Bioactive Compounds

Fifteen lettuces of each treatment and variety were stored in refrigeration (4–8 °C). The stability experiment was carried out for three weeks, and each week, a group of 5 lettuces per treatment was taken, frozen, and lyophilized. At the end of the experiment, the total polyphenols, total anthocyanins (only in red lettuce), and total carotenoids were quantified.

#### 2.2.1. Extraction and Quantification of Polyphenols

Polyphenols were extracted from green and red lettuce samples with 80% (*v*/*v*) methanol in water, and the total phenolic content (TPC) was determined by the Folin–Ciocalteu method as previously reported [5]. Results were expressed as mg of gallic acid equivalents per g of dry sample (mg GAE/g DW).

#### 2.2.2. Extraction and Quantification of Total Anthocyanins

The total anthocyanins (TAN) were extracted and quantified only in red lettuce using the method developed by Lee et al. [14], modified to apply it to lettuce by Moreno-Escamilla et al. [5]. Absorbance was measured at 520 and 700 nm on a microplate after 30 min of incubation at room temperature. A value for absorbance (A) was calculated using Equation (1).

A = (A520 nm_pH 1.0_ − A700 nm_pH 1.0_) − (A520 nm_pH 4.5_ − A700 nm_pH 4.5_) (1)

The total anthocyanin content was calculated using Equation (2). Results were expressed as mg of cyanidin 3-rutinoside/g of the dry sample (mg Cy3Rt/g DW).

Total anthocyanins = (A × 449.2 × 25 × 1000)/(26900)(2)

#### 2.2.3. Extraction and Quantification of Total Carotenoids

Total carotenoids (CARs) were quantified in green and red lettuce after extracting samples with acetone [15]. Fifty milligrams of freeze-dried samples were mixed with 10 mL of acetone, sonicated for 20 min (40 KHz) at room temperature (25 °C), and centrifuged at 2000× *g* for 10 min (4 °C). The supernatant was collected, and the residue was re-extracted twice under the same conditions. Supernatants were combined, 250 μL was placed on a microplate, and absorbance was read at 474 nm. Total carotenoids were determined using Equation (3):

mg β-carotene/mg sample = (A × V × DF × 10)/(g × E1% cm)(3)

where A = absorbance, V = volume (30 mL), DF = dilution factor, g = grams of sample, and E1% cm = the specific extinction coefficient of β-carotene, which is 2500 [16]. Results were expressed in mg β-carotene/g of dry sample (mg βCar/g DW).

### 2.3. Sensory Evaluation

Green and red lettuce samples treated with elicitors were subjected to a descriptive sensory analysis through a trained panel of nine judges. Both control and elicitor-treated samples were evaluated to characterize their main sensory attributes. All sensory analyses were carried out within the first week of storage. Descriptive analysis was performed using the Spectrum^®^ method [17] with some modifications. Attributes were evaluated in five phases: (i) olfactory phase (smell: general intensity, first impression, and descriptors were determined by a focus group technique, (ii) taste (sweet, bitter, sour), (iii) tactile in the mouth (astringency, wettability, and crispiness), (iv) tactile (firmness) and (v) visual phase (color intensity and brightness). The judges evaluated each attribute using a 150 mm linear scale labeled “Not at all…” and “Extremely…” at either end. Each judge marked the scale line according to the intensity with which each attribute or descriptor was perceived in each sample. The distance between the left end of the line and the judge’s mark was measured and registered as the intensity level perceived for each attribute in each sample. All tests were conducted in individual boots. Before evaluating each sample, each judge was presented, in duplicate, with three randomized standards corresponding to each attribute to stabilize the use of the scale at a low, medium, and high point. The design with which each attribute or descriptor test was presented to the panel was completely randomized, balanced, and counterbalanced, and each evaluation was performed in duplicate. The panel used eye covers in all tests except for the visual phase. There were ten work sessions for the panel, each lasting 60 min and with breaks of 10 min between one test and another. A maximum of two attributes or descriptors were evaluated per session. The lettuce samples were removed from refrigeration no longer than 30 min before the tests to maintain the integrity of the lettuce leaf. On the evaluation day, the lettuce samples were cut using one leaf to analyze each treatment. For the taste and oral tactile tests, samples of butterhead lettuce leaves were washed and sanitized (200 ppm solution of commercial chlorine for 15 s). The samples were placed in a plastic dish, identified with random three-digit numbers, and given to the judges for evaluation. The judges rinsed their mouths with purified water (Alaska^®^, Chihuahua, México) at the beginning and between samples in each test. Each judge received a response sheet for each attribute or descriptor [18]. An example of a response sheet is provided in Supplementary Materials (Figure S1).

### 2.4. Statistical Analysis

For phytochemical stability, a pool of all individuals for each treatment (5 lettuces) was made to minimize the variability among individual samples. All analyses were carried out in triplicate, and a two-way ANOVA with Tukey’s multiple comparisons was performed to determine statistical differences between elicitors and the time of storage. Sensory profile data were analyzed using repeated measures analysis of variance (ANOVA) with Fisher’s multiple comparisons (LSD). All the analyses were carried out using the statistical program XLSTAT version 2016.05 (Addinsoft^®^, Paris, France). Values are expressed as the mean ± standard deviation (SD). The criterion for statistical significance was *p* < 0.05.

## 3. Results and Discussion

### 3.1. Shelf-Life Stability of Bioactive Compounds

Previous results obtained by our research group [5,6] showed that using stress response elicitors was a viable strategy to increase the content of phenolic compounds (including anthocyanins) and carotenoids in green and red butterhead lettuce. The present work evaluated the stability of these bioactive molecules during the lettuce shelf life (3 weeks, according to the producer).

**Figure 1:**
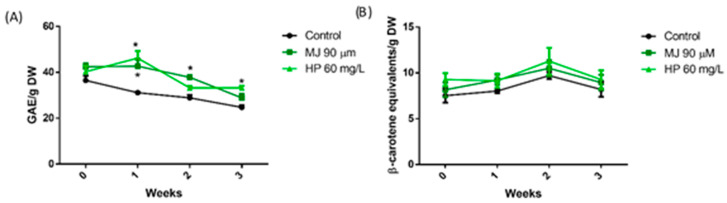
Concentration of bioactive phytochemicals during a 3-week storage period of green butterhead lettuce samples treated with elicitors. Concentration of total phenolic compounds (**A**) and total carotenoids (**B**). * represents an increase with a significant difference compared to the control of the corresponding week (*p* < 0.05).

Figure 1 shows the total phenolic compounds (TPC) and carotenoid (CAR) behavior during the shelf-life of green lettuce. The concentration of TPC in control samples decreased through storage time with a loss of 32% of the initial content, and treatment with both elicitors reduced the loss in the TPC. At harvest time (week 0), a slight non-significant increase in the TPC was observed in elicitor-treated samples. A further increase was observed after one week of storage, so the TPCs were significantly higher in the samples treated with MJ and HP than in control samples. At week two, both treatments suffered a decrease in the TPC concentration by 10 and 20% for MJ and HP, respectively; however, the TPC in MJ-treated samples was still significantly higher than the control. At week three, the TPCs were maintained at levels similar to those in week two in HP-treated samples. It is worth mentioning that samples at week three presented visual symptoms of decay, so it is suggested that the ideal shelf-life of green lettuce should be two weeks, the time in which the samples maintained their integrity, as well as a high concentration of bioactive phytochemicals. On the other hand, the CAR content in green lettuce (Figure 1B) was not modified in response to any treatments or storage time. In other words, carotenoids in green lettuce were more stable than phenolic compounds during storage, and, contrary to phenolic compounds, carotenoids did not respond to elicitors.

**Figure 2:**
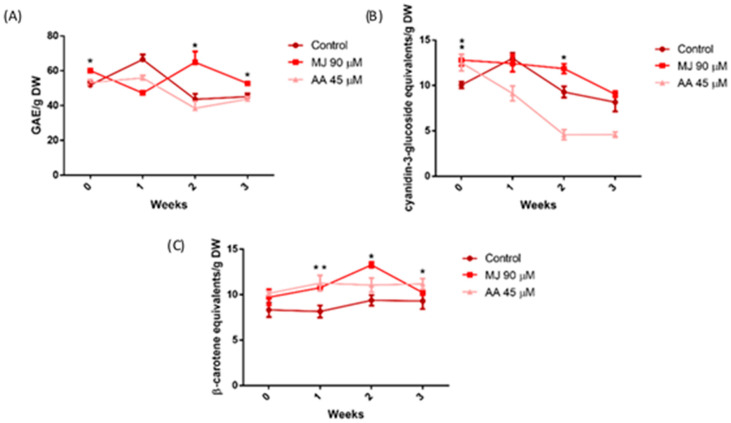
Concentration of phytochemicals during a storage period of red butterhead lettuce samples treated with elicitors. Concentration of total phenolic compounds (**A**), total carotenoids (**B**), and anthocyanins (**C**). * represents an increase with a significant difference compared to the control of the corresponding week (*p* < 0.05). ** an increase with a significant difference compared to the control of the corresponding week (*p* < 0.01).

Figure 2 shows the behavior of the TPC, CAR, and total anthocyanins (TAN) during the shelf life of red butterhead lettuce. Anthocyanins are a family of flavonoids commonly found in red and purple fruits and vegetables; they are present in red butterhead lettuce and are responsive to elicitation [6]. Unlike green butterhead lettuce, control samples of red butterhead lettuce did not show a significant decrease in their TPC during the 3-week storage. Treatment with AA (45 µM) had no effect, so the TPC of AA-treated samples remained the same as in the control in all storage times. In contrast, samples treated with MJ (90 µM) showed significantly higher levels of TPC on weeks 0 (immediately after harvest), 2, and 3 (Figure 2A). In the case of the TAN, they behaved like the TPC, with no significant decrease in control samples at the end of the storage period. Nevertheless, a significant increase in the TAN was observed in week 0 for both elicitors, although AA-treated samples showed a significant and steady decrease (more than 40% in the first week) during the storage period, so the TAN levels were lower than in the control in AA-treated samples in weeks 1–3. On the other hand, the anthocyanin content in the MJ-treated samples remained constant until week 2, when it was significantly higher than the control, and then decreased in week 3 to values similar to the control (Figure 2B). The CAR content in red lettuce samples remained constant throughout storage for the control samples, similarly to the results in green lettuce. At week 0, neither elicitor showed a significant effect; however, a slight increase in the CAR content was observed for both elicitors at week 1, so the CAR content was significantly higher in elicitor-treated samples at this sampling time. At week 2, MJ elicited a further increment in the CAR content, which reached their highest levels in this storage time. At week 3, the CAR content decreased in MJ-treated samples to levels similar to the control, while in AA-treated samples, it remained higher than the control. The visual inspection of red lettuce did not show apparent symptoms of decay, as the green lettuce did. Nevertheless, the highest content of bioactive compounds was observed at week 2 in MJ-treated samples, so two weeks could be considered an optimal shelf-life for both lettuce varieties.

In summary, the effect of elicitors varied with the time of sampling, type of phytochemical, and lettuce type. In green lettuce, MJ and HP showed a clear tendency to increase the content and stability of the TPC but did not affect the CAR. In red lettuce, MJ had the best effects, increasing the TPC and TAN at 0 and 2 weeks of storage and the content of CAR at weeks 1 and 2. Red lettuce samples treated with AA showed a marked decrease in TAN stability during the 3-week storage period.

Studies carried out in fruits and vegetables to evaluate changes in bioactive metabolites in response to elicitor treatments have usually shown elevation in TPCs. Phenolic compounds are known to act as chemical defenses against several biotic and abiotic stresses, so stress response elicitors usually activate the metabolic synthesis of these compounds by triggering key enzymes of the phenylpropanoid pathway, such as phenylalanine ammonia-lyase (PAL) [8]. In one study, the addition of MJ to romaine lettuce during a short period (7 days) showed an initial increase in the TPC, followed by a decrease in subsequent days. The authors established that plants can respond quickly to stress, but this effect is short-lasting due to their low stability [19]. The present results are somewhat different since the TPC decreased during storage in green but not in red butterhead lettuce, and MJ induced a modest but sustained increase in these compounds in both types of lettuce. The present results are also different from those of Zlotek et al. [12], who found that AA (100 µM) was the best elicitor for increasing the content of bioactive compounds in butterhead lettuce. However, those authors did not use MJ or HP as treatments, and a previous study by our research group found modest effects of AA compared to MJ and HP [6], which was confirmed by the present results. MJ was also the best treatment when individual phenolic compounds were analyzed by HPLC-MS in the previous study; the compounds increased by MJ belonged to the classes of flavonoids (flavonols such as quercetin and kaempferol glycosides) and hydroxycinnamic acids (caffeic acid derivatives) [6]. MJ also elicited the activation of key enzymes of the phenylpropanoid pathway, such as PAL and CHS (chalcone synthase) [6]. HP showed effects comparable to those of MJ in green butterhead lettuce, which agrees with the results from our previous paper [6]. HP is a bacterial protein that elicits systemic acquired resistance in plants. It has been reported that HP activates a salicylic acid-dependent, methyl jasmonate-independent pathway in *Arabidopsis thaliana* [20]. However, more recent works have suggested the activation of multiple defense signaling pathways, including the methyl jasmonate pathway, in soybean [21]. To our knowledge, the mechanism of action of HP has not been studied in lettuce, but it could involve an increase in MJ, which in turn could activate the phenylpropanoid pathway and increase the content of flavonoids and hydroxycinnamic acids. Further studies should be carried out to test this hypothesis.

Anthocyanins are a family of flavonoids; they are also synthesized through the phenylpropanoid pathway, so several elicitors are known to increase their content in red- and purple-colored plants. For example, the effect of MJ on the stimulation of TAN synthesis has been reported in grapes [22] and radishes [23], which agrees with the results observed in the present work in red lettuce. Two studies have analyzed the effect of nutrient deprivation on the content of anthocyanins and other bioactive compounds in red butterhead lettuce. In both works, nutrient eustress (moderate deprivation) increased the anthocyanin content, as well as the content of other specific phenolic compounds, and red cultivars were more responsive to nutrient eustress [24,25], which is similar to the present results, using MJ as an abiotic stressor. Other authors have mentioned the susceptibility of anthocyanins to oxidation [26]; however, in the present investigation, the TAN only decreased in AA-treated red lettuce, indicating a possible role of AA in anthocyanin degradation. Dedyukhina et al. [27] reported that AA concentrations above 10^−5^ M induce necrosis in plant tissues, and considerably lower concentrations (10^−7^ M) are required to elicit systemic defense response. The AA concentration used in the present work was 4.5 × 10^−5^ M, which could explain the deleterious effects on anthocyanins during red lettuce storage. Nevertheless, a previous work by our research group [6] found no undesirable effects of the preharvest application of 45 µM (4.5 × 10^−5^ M) and up to 90 µM of AA. These new results indicate a possible adverse effect of AA on the shelf-life of butterhead lettuce and highlight the importance of analyzing both pre- and post-harvest changes in bioactive compounds in plant foods. Growth and stress regulators have also been used as post-harvest treatments that increase the content of bioactive compounds and antioxidant activity of plant foods [3,28].

Fewer studies have addressed the effect of stress elicitors on the CAR content. Carotenoids are a diverse group of isoprenoids derived from the linear tetraterpene phytoene synthesized by the enzyme phytoene synthase, considered a crucial enzyme for carotenoid biosynthesis [29]. An increase in CAR in response to stress elicitors and their combinations was observed in bell pepper samples [30]; in another study, elicitation with salicylic acid and MJ significantly increased the carotenoid content in moringa samples through the activation of lycopene-β-cyclase, another enzyme central to carotenoid metabolism [31]. In a previous study by our research group, CARs were increased in green and red butterhead lettuce by treatment with MJ, HP, and AA. The treatments also stimulated the activity of the enzyme lycopene β-cyclase [6]. In contrast, in the present investigation, the CAR contents were only increased in red lettuce by treatment with MJ and only after one and two weeks of post-harvest storage. On the other hand, carotenoids were stable throughout storage in both lettuce types. Carotenoids are mainly recognized for their role as accessory pigments in photosynthesis, but some studies have also found that they have a role in plant stress response and growth regulation, especially secondary carotenoids such as apocarotenoids and others [29]. The present results suggest that carotenoids have a minor role compared to phenolic compounds as mediators of stress response in butterhead lettuce; however, the role and identity of the carotenoids involved in stress response in lettuce must be further studied.

### 3.2. Sensory Characterization

Results described in the previous section, as well as previously published works, have shown that the treatment of green and red butterhead lettuce with stress response elicitors (MJ 90 µM and HP 60 mg/L for green, and MJ 90 µM for red) increased the content of phenolic compounds and, to a lesser degree, of carotenoids. Phenolic compounds act as defense compounds in the plant; however, they can also modify the organoleptic characteristics of the edible parts and may be the leading cause of the bitter taste of foods [32]. In the same way, carotenoids are the main molecules responsible for the tones or intensities of color in lettuce leaves, so they can impact the color and taste that can be perceived in the lettuce samples [33]. Therefore, it is necessary to establish if the increase in these compounds substantially affected the organoleptic characteristics of treated lettuce.

**Figure 3:**
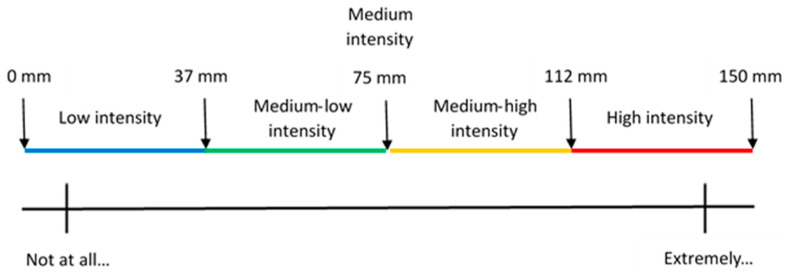
Scale used by the trained judges to evaluate the intensity levels of each attribute in each sample (see Section 2 and Supplementary Materials for more details).

In this research, the sensory characterization was carried out using a descriptive sensory analysis performed by a trained panel of nine judges. A total of 15 attributes and descriptors were perceived and evaluated by human senses, such as smell, taste, touch (tactile and tactile in mouth), and sight (Table 1). The intensity of each attribute was evaluated by each judge in each sample (control and elicitor-treated) with a 150 mm scale from “Not at all [attribute]” to “Extremely [attribute]” (Figure 3). For a better interpretation, the scale was divided into five intensity levels: low (L, 0 to 37 mm), medium-low (ML, 38 to 74 mm), medium (M, 75 mm), medium-high (MH, 76 to 112 mm), and high (H, 113 to 150 mm) (Figure 3). Despite minor differences in the numerical values of the perceived intensities of the attributes among treatments (See Supplementary Materials Tables S1 and S2), no significant differences were observed between the treatments and control in all the evaluated attributes in both types of lettuce, indicating no effect of elicitors on the sensory attributes of butterhead lettuce.

**Figure 4:**
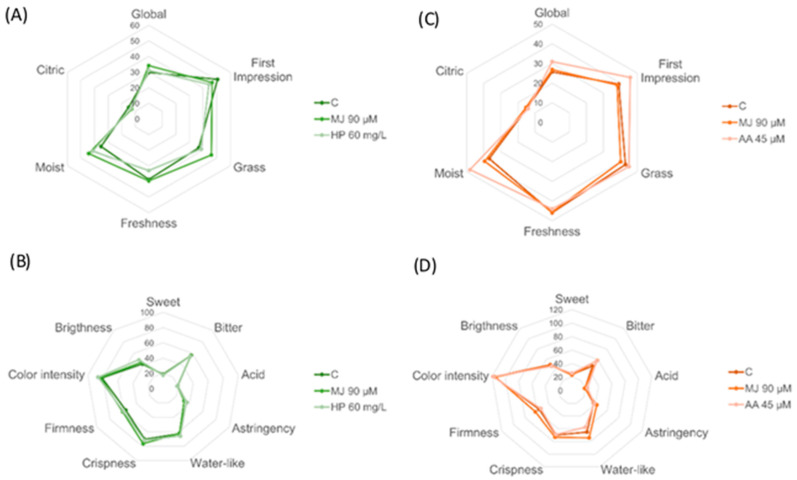
Sensory profile of butterhead lettuce treated with and without elicitors (C, control; MJ, methyl jasmonate; HP, Harpin protein; AA, arachidonic acid). (**A**,**C**), olfactory descriptors for green and red lettuce, respectively. (**B**,**D**), sensory attributes of taste, oral tactile, tactile, and sight phases, for green and red lettuce, respectively.

Since no significant differences were found among treatments, the perceived intensities of each attribute were averaged to better understand the sensory profile of butterhead lettuce (Table 1). According to this scale, both types of lettuce showed the same profile of aroma and flavor attributes, characterized by L and ML intensity levels. Green and red butterhead lettuce showed an L odor intensity and ML first impression (odor when tearing the plant tissue). The odor descriptors identified by the panel for both types of lettuce were grass, freshness, moist, and citrus. Grass, freshness, and moist were perceived with similar intensities (ML), while the citrus aroma was perceived with a L intensity. Figure 4A,C show the aroma profile of green and red lettuce with and without elicitor treatment. As mentioned above, none of the stress elicitors induced any significant change in the sensory attributes, and the profiles of both lettuce varieties were similar. However, in red lettuce, AA tended to increase the intensity of the global, first impression, and moist aroma. In green lettuce, MJ showed a slight tendency to increase the grass aroma.

In taste attributes, bitter was perceived with the highest intensity (ML in both lettuce varieties), while sour and sweet were both L (Table 1). In fact, bitter was the most prominent attribute in both the flavor and aroma phases. Bitterness is a flavor attribute that has been related to the presence of phenolic compounds [32]; nevertheless, other less known phytochemicals have also been related to this flavor in lettuce. Sesquiterpene lactones are a class of terpenoids found in some plant families, especially in *Asteraceae* (where the lettuce species belongs); they are usually conjugated with oxalate and sulfate, are released in response to various stressors, and have been linked to the bitterness of different lettuce cultivars [3]. Further studies should aim to identify these compounds in butterhead lettuce since they may also possess bioactive properties [3].

Food sensory evaluation also deals with the tactile and visual attributes of the food products. In the present work, oral tactile (astringency, water-like or wettability, and crispness), tactile (firmness), and visual or sight (color intensity and brightness) attributes of green and red lettuce were evaluated (Table 1). For oral tactile attributes, green and red lettuce showed the same level (ML) of crispiness and wettability. At the same time, astringency was perceived with a higher intensity in red (ML) than in green (L) lettuce. Crispiness was one of the attributes perceived with a higher intensity; it was ML, on average, on both lettuce varieties, but its values (70.7 to 72.7 mm) were very close to the M level (see Figure 4) and higher than all aroma, flavor, and other tactile attributes. In the tactile phase, firmness was the only attribute evaluated and it showed a ML intensity in both lettuce varieties. Color intensity, one sight attribute, was perceived with the highest intensity in comparison with all the other evaluated attributes; it was MH in green lettuce and H in red lettuce. Brightness was perceived as ML in both lettuce types. In summary, color intensity was the most prominent attribute of both types of lettuce and was perceived with a higher intensity in the red samples; crispness and wet-like feeling (wettability), both oral tactile attributes, were also outstanding. These three attributes showed higher perceived intensity values than the primary odor and taste attributes (bitter taste).

In agreement with the results described for odor attributes, flavor, tactile, tactile in the mouth, and visual attributes were not significantly affected by elicitor treatment in green or red lettuce (Figure 4B,D). However, AA showed a minor effect in red lettuce, reducing the perceived wet-like feeling, while MJ caused a minor increase in the same attribute. This Figure also shows that the most prominent attributes in the sensory profile of both lettuce varieties were color intensity, crispness, wettability, and bitter flavor.

In summary, our results indicate that the use of MJ 90 µM and HP 60 mg/L, applied seven days before harvest in green butterhead lettuce, and MJ 90 µM and AA 45 µM, applied 15 days before harvest in red butterhead lettuce, did not affect the organoleptic characteristics of these vegetables. Little research evidence has been found in which the effect of stress response elicitors on the organoleptic characteristics of lettuce samples has been investigated, and it mostly agrees with the results obtained in this investigation. Złotek et al. [12] evaluated the effect of AA, jasmonic acid, and abscisic acid in green butterhead lettuce samples and did not observe any significant effect of elicitors on the appearance, color, and aroma. In the same way, treatment with chitosan and tea tree essential oil did not induce any alterations in visual quality parameters (leaf texture, leaf color, leaf brightness, leaf browning, and browning at the cut base) in butterhead lettuce samples [34]. Nevertheless, none of those studies conducted a descriptive analysis in which the intensities of different sensory attributes were evaluated, as in the present investigation.

Results described in the previous section and previously published data showed an increase of approximately 10–20% of phenolic compounds in green and red lettuce leaves. Phenolic compounds are known to impact some sensory properties of food, such as texture, color, and taste. In the case of taste, they can promote the presence of bitterness in the foods, where they are found through the activation of receptors (TAS2R) coupled to G proteins [32,35]. The bitter taste and color intensity were some of the main attributes in the samples, and they remained unchanged despite the increase in phenolic compounds. Moreover, in red lettuce treated with AA, the TAN decreased with storage time; however, the panel perceived no change in color intensity or brightness (which are usually dependent on the anthocyanin content). Both observations could be explained according to Stevens’ Law, which establishes that the intensity in the perception of a stimulus is a function of the magnitude of the physical stimulus, particularly in suprathreshold conditions [36]. Then, it can be inferred that the increase in phenolic compounds or decrease in anthocyanin, observed in this research are not large enough to modify the intensity of perception by the judges. These results allow us to suggest that the use of stress response inducers in green (MJ 90 µM and HP 60 mg/L) and red (MJ 90 µM and AA 45 µM) butterhead lettuce samples can significantly change the content of bioactive phytochemicals without modifying the sensory attributes of the product.

## 4. Conclusions

Stress response elicitors induced changes in bioactive compounds of green and red butterhead lettuce without modifying the organoleptic properties of the samples, preserving the original characteristics of butterhead lettuce, such as color intensity, crispness, wettability, and bitter taste. In general, all bioactive compounds showed the highest levels at week 2 of storage, so a 2-week shelf life is recommended for these varieties of butterhead lettuce. MJ 90 µM was the best treatment since it increased the TPC throughout storage at 4 °C (especially during the first two weeks) in green and red lettuce and the TAN and CAR content in red lettuce. HP 60 mg/mL and AA 45 µM also showed some effects on the content of bioactive compounds in green and red butterhead lettuce. Together, these results conclude that the use of elicitors in adequate concentrations may significantly increase the content and stability of bioactive metabolites without modifying the food sensory properties, suggesting that this technique can be recommended for use by producers to increase the potentially functional value of green and red butterhead lettuce grown in hydroponic conditions.

## Figures and Tables

**Table 1:** Definition and average perceived intensity of the attributes evaluated by the trained judges for the descriptive sensory analysis.

	Attribute	Definition	Average Perceived Intensity (in mm) and Level of Intensity *	
			Green Lettuce	Red Lettuce
Aroma	Global	Strength of the overall perception	31.7 ± 2.2 L	27.7 ± 2.7 L
	First impression	Perception of aroma after cutting the leaf	47.2 ± 3.2 ML	40.9 ± 4.2 ML
	Grass	The aromatics associated with dried grass or hay	40.5 ± 4.9 ML	42.6 ± 2.5 ML
	Freshness	Related to green leaves notes	37.1 ± 3.6 ML	45.1 ± 1.1 ML
	Moist	Related to water notes	40.2 ± 4.6 ML	41.5 ± 5.9 ML
	Citric	Related to citric acid notes	13.5 ± 1.1 L	14.6 ± 0.6 L
Flavor	Sweet Bitter Sour	Sensation related to different sugar solutions Sensation related to different caffeine solutions Sensation related to different citric acid solutions	18.0 ± 0.7 L 56.0 ± 0.7 ML 18.9 ± 0.3 L	24.3 ± 1.4 L 54.2 ± 6.2 ML 20.6 ± 2.3 L
	Astringency	The shrinking or puckering of the tongue surface	33.9 ± 1.9 L	39.1 ± 2.9 ML
Oral tactile	Water-like	The amount of moisture in the sample	63.4 ± 1.9 ML	65.6 ± 8.3 ML
	Crispness	The force with which lettuce crumbles	72.7 ± 3.1 ML	70.7 ± 2.3 ML
Tactile	Firmness	The force required to compress the lettuce sample	59.0 ± 2.4 ML	57.2 ± 4.8 ML
Sight	Color intensity Brightness	The intensity or strength of the color from light to dark The intensity of reflecting light.	83.5 ± 2.9 MH 45.4 ± 3.5 ML	116.9 ± 1.7 H 48.4 ± 1.5 ML

* Levels of intensity: L, low; ML, medium low; M, medium; MH, medium high; H, high.

## Data Availability

The data presented in this study are available on request from the corresponding author due to privacy issues.

## Supplementary Materials

The following supporting information can be downloaded at: https://www.mdpi.com/article/10.3390/foods13162574/s1, Figure S1. Response sheet used by the trained panel for the descriptive sensory analysis. Table S1. Main attributes and descriptors of aroma and flavor evaluated in samples of green and red butterhead lettuce treated with elicitors. Values are in mm, of a linear scale of 150 mm. Table S2. Main oral tactile, tactile and visual attributes and descriptors evaluated in samples of green and red butterhead lettuce treated with elicitors. Values are in mm, of a linear scale of 150 mm.
