# Supplementary material for: The Preharvest Application of Stress Response Elicitors Improves the Content of Bioactive Compounds without Modifying the Sensory Attributes of Butterhead Lettuce (Lactuca sativa var. capitata)
Source: Foods. 2024 Aug 17;13(16):2574. doi: 10.3390/foods13162574 (PMC11353814; doi:10.3390/foods13162574)
Supplement: Supplementary file 1 [file foods-13-02574-s001.zip › foods-3125121-supplementary.pdf]

**Preharvest application of stress response elicitors improves the content of bioactive compounds without modifying the sensory attributes of butterhead lettuce (*Lactuca sativa* var. *capitata*).**

**Supplementary Material.**

**Table S1.** Main attributes and descriptors of aroma and flavor evaluated in samples of green and red butterhead lettuce treated with elicitors. Values are in mm, of a linear scale of 150 mm.

| Sample | Elicitor | Aroma | | | | | | Flavor | | |
|---|---|---|---|---|---|---|---|---|---|---|
| | | Global | First impression | Grass | Freshness | Moist | Citric | Sweet | Bitter | Sour |
| Green Lettuce | C | 29.8 ± 11.8[a] | 50.7 ± 11.3[a] | 36.9 ± 18.3[a] | 38.6 ± 11[a] | 35.2 ± 14.8[a] | 14.8 ± 6.4[a] | 18.8 ± 11[a] | 56.1 ± 20.5[a] | 19.2 ± 10.5[a] |
| | MJ 90 µM | 34.1 ± 12.2[a] | 46.7 ± 12.7[a] | 46.1 ± 21.4[a] | 39.6 ± 11.8[a] | 44.2 ± 20.9[a] | 12.6 ± 2.9[a] | 17.8 ± 8.3[a] | 56.7 ± 12.7 [a] | 18.8 ± 9.4[a] |
| | HP 60 mg/L | 31.1 ± 17.3[a] | 44.3 ± 13.7[a] | 38.5 ± 15.6[a] | 33 ± 6.3[a] | 41.3 ± 21[a] | 13.2 ± 4.3[a] | 17.5 ±7.3[a] | 55.3 ± 12. 5[a] | 18.6 ± 9.5[a] |
| Red Lettuce | C | 25.6 ± 9.9[a] | 39 ± 14.6[a] | 42.9 ±15.5[a] | 45.9 ± 21.6[a] | 36.8 ± 17.4[a] | 14.6 ± 6.2[a] | 24.1 ± 14.1[a] | 47.1 ± 22.9[a] | 19.5 ± 9.5[a] |
| | MJ 90 µM | 26.8 ± 8.6[a] | 38.1 ± 14.2[a] | 40.1 ± 8.5[a] | 45.5 ± 14.7[a] | 39.5 ± 20[a] | 15.2 ± 7.6[a] | 23 ± 12.3[a] | 58 ± 23.3[a] | 19 ± 12[a] |
| | AA 45 µM | 30.8 ± 13.3[a] | 45.7 ± 13.5[a] | 44.9 ± 21.3[a] | 43.9 ± 20.5[a] | 48.1 ± 25.5[a] | 14 ± 5.4[a] | 25.7 ± 15[a] | 57.6 ± 26.4[a] | 23.2 ± 13.5[a] |

Mean values ± SD. AA, arachidonic acid; C, control; MJ, methyl jasmonate; HP, Harpin protein. Comparison between treatments in the same attribute or descriptor in the same variety. Statistical significance at $p < 0.05$.

**Table S2.** Main oral tactile, tactile and visual attributes and descriptors evaluated in samples of green and red butterhead lettuce treated with elicitors. Values are in mm, of a linear scale of 150 mm.

| Sample | Elicitor | Oral tactile | | | Tactile | Sight | |
|---|---|---|---|---|---|---|---|
| | | Astringency | Water-like | Crispness | Firmness | Color intensity | Brightness |
| Green Lettuce | C | 35.2 ± 21.6[a] | 62.5 ± 22.8[a] | 70.2 ± 27.3[a] | 56.3 ± 21.6[a] | 80.8 ± 9[a] | 42.2 ± 17.3[a] |
| | MJ 90 µM | 31.7 ± 23.7[a] | 62.2 ± 28.2[a] | 76.2 ± 28.3[a] | 61 ± 23.5[a] | 83.3 ± 10.4[a] | 44.8 ±14.1[a] |
| | HP 60 mg/L | 34.9 ± 17.1[a] | 65.6 ± 23.2[a] | 71.7 ± 27[a] | 59.6 ± 17.3[a] | 86.5 ± 14.7[a] | 49.1 ± 15.8[a] |
| Red Lettuce | C | 37.1 ± 18.6[a] | 65.2 ± 19.7[a] | 70 ± 23.3[a] | 55.7 ± 14.8[a] | 117 ± 10.6[a] | 49.8 ± 15.3[a] |
| | MJ 90 µM | 42.4 ± 23.3[a] | 74.1 ± 24.5[a] | 73.2 ± 26.2[a] | 62.6 ± 15.1[a] | 115.1 ± 18.4[a] | 48.4 ± 16.6[a] |
| | AA 45 µM | 37.9 ± 15.1[a] | 57.6 ± 23.9[a] | 68.8 ± 25.1[a] | 53.3 ± 14.8[a] | 118.5 ± 18.8[a] | 46.9 ± 12.9[a] |

Mean values ± SD. AA, arachidonic acid; C, control; MJ, methyl jasmonate; HP, Harpin protein. Comparison between treatments in the same attribute or descriptor in the same variety. Statistical significance at $p < 0.05$.

**Figure S1**

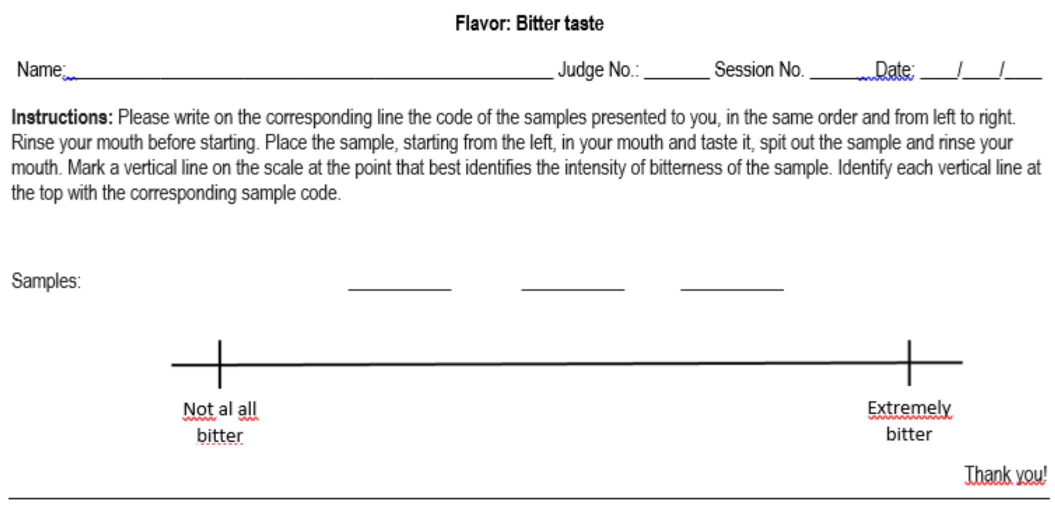

**Flavor: Bitter taste**

Name:________________________________________ Judge No.: _______ Session No. _______ Date: ____/____/____

**Instructions:** Please write on the corresponding line the code of the samples presented to you, in the same order and from left to right. Rinse your mouth before starting. Place the sample, starting from the left, in your mouth and taste it, spit out the sample and rinse your mouth. Mark a vertical line on the scale at the point that best identifies the intensity of bitterness of the sample. Identify each vertical line at the top with the corresponding sample code.

Samples: __________ __________ __________

Not al all bitter

Extremely bitter

Thank you!

**Figure S1.** Response sheet used by the trained panel for the descriptive sensory analysis.
